# Editorial: Environmental clues to brain disease

**DOI:** 10.3389/fnagi.2023.1220484

**Published:** 2023-06-13

**Authors:** Patrizia Giannoni, Federica Bertaso, Gustavo Provensi

**Affiliations:** ^1^UPR CHROME (Risques CHROnique et eMErgents), University of Nîmes, Nîmes, France; ^2^IGF, University of Montpellier, CNRS, Inserm, Montpellier, France; ^3^Department of Neuroscience, Psychology, Drug Research and Child Health (NEUROFARBA), University of Florence, Florence, Italy

**Keywords:** inflammation, excitotoxicity, precision medicine, circadian rhythm, contaminants

Brain disease represents one of the major social and economic burden worldwide. Identifying the factors impacting brain homeostasis, as well as the mechanisms involved in this deregulation, constitutes a paramount research challenge. If several pathologies have been associated to specific genetic mutations, many other elements contribute to the development of a broad panel of brain diseases. In this Research Topic we collected articles that are analyzing ground-breaking subjects shedding light on the interaction between environment and brain health. Here is a summary of the main findings highlighted in the topic.

## Genetic polymorphism and Parkinson's disease risk

For the first time, Fan et al. found an association between the polymorphism of Cytochromes P450 (CYPs), Glutathione S-transferases (GSTs) and Parkinson's disease (PD) development linked to the exposure to xenobiotics, including drugs and pesticides. The authors analyzed 13 PD-associated polymorphisms in genes encoding metabolizing enzymes and found that none of them resulted associated to PD when analyzed independently. On the contrary, the synergistic association of multiple polymorphisms concerning CYPs and GSTs, but not esterases, is linked to the modulation of PD risk. An interesting point discussed by the authors is that the expression of GSTs might be regulated by sex hormones, explaining at least partially the female-specific effect that has been observed.

## Sex and brain disease

Indeed, the link between brain disease and sex has been studied for a long time, but many questions remain unanswered. While women's greater longevity compared to men has been considered responsible for the higher risk of AD, we now know that exposure to estrogens in life plays a key role. Several studies focused on parameters such as reproductive lifespan, menopause status, number of pregnancies, exposure to hormonal therapy (HT) or hormonal contraceptive (HC), but also on anti-estrogen treatment as for example in the case of hormonal-dependent cancers. Jett et al. summarized the main findings of these studies and concluded that cumulative estrogen exposure in life seems to have a supportive role of neurological health. The authors underlined that additional studies including neuroimaging biomarkers and longer follow-up periods are necessary, as well as larger and more diverse samples. A one-size-fits-all approach in preventive care seems not applicable and personalized evaluation of therapies should be considered by physicians. For example, hormonal therapy has been suggested as protective against cerebrovascular and metabolic aging if applied in midlife, while the effects might be exactly opposite if started more than 5 years after menopause. Only a fine-tuned analysis of a patient's history will lead to an effective precision medicine.

## Circadian rhythm on Alzheimer's disease-related alterations

Other factors that have been studied in the topic include the impact of circadian rhythm on AD and, more generally, on memory functions. Da Silva et al. compared an AD model, the 3x-tg mice with WT mice and evidenced that the transgenic model has an altered circadian pattern concerning multiple memory-related tasks. Morris water maze and reversal spatial learning tasks evidenced an altered circadian variation in hippocampus-dependent learning in the AD model. In addition, cerebral cortical synaptosomes showed a circadian variation of hippocampal long-term potentiation, FCCP-stimulated oxygen consumption and mitochondrial calcium retention blunted in transgenic mice when compared to WT. This pioneering study suggests that the archetypical alterations of mitochondrial activity, synaptic plasticity, memory performance observed in AD might all be under the control of the circadian rhythm.

## Zinc serum levels and Parkinson's disease-associated dementia

Life-style is globally pointed out as a major contributor to brain disease risk. The study of Lee et al. addressed the contribution of zinc serum levels to non-motor symptoms associated to PD, with a special focus on dementia. While excessive exposure to heavy metals has been associated to detrimental effects such as oxidative stress, some of them are, depending on the concentrations, fundamental for proper enzyme functioning. This retrospective cohort study linked low serum zinc levels to PD-associated dementia (PD-D) and suggested zinc levels as a biomarker for the prediction of PD-D conversion.

### Astrocytes redox status and protection from oxidative stress

Hormetic mechanisms describing adaptative changes to environmental stimuli are also the focus of a recent article by Mokrane et al.. The authors underline the fundamental role of astrocytes in regulating brain homeostasis via the production of glutathione, a primary antioxidant. Through the use of astroglioma cells, they were able to demonstrate how changes in the redox status of astrocytes bring to upregulated Gq-linked GPCR responses via P2Y receptors. The authors conclude that deficits in GSH, that we know are associated to neurodegenerative and neurodevelopmental diseases, might bring to changes in the redox state of astrocytes and finally contribute to altered brain homeostasis.

Taken together, this collection underlines how brain disease is the result of a plethora of parameters that are intertwined and contribute to different extents and via different type of interactions (e.g., summative or synergistic) to the development of pathological pathways. Only large-scale studies and the worldwide sharing of data sets will allow a better understanding of each contribution and help to the development of a precision medicine and a personalized preventive care.

## Author contributions

PG prepared the original draft which was edited by FB and GP. All authors have read the final version of the manuscript and approved its publication.

